# Experimental and Numerical Analysis of the Strain Response of Additively Manufactured Composites

**DOI:** 10.3390/polym18212697

**Published:** 2026-10-31

**Authors:** Miroslav Pástor, Pavol Lengvarský, Jozef Svetlík

**Affiliations:** 1 Department of Applied Mechanics and Mechanical Engineering, Faculty of Mechanical Engineering, Technical University of Košice, Letná 1/9, 042 00 Košice-Sever, Slovakia; miroslav.pastor@tuke.sk; 2 Department of Production Systems and Robotics, Faculty of Mechanical Engineering, Technical University of Košice, Letná 1/9, 042 00 Košice-Sever, Slovakia; jozef.svetlik@tuke.sk

**Keywords:** Onyx, fiberglass, strain gauge, finite element analysis

## Abstract

This paper investigates the effect of reinforcement layer configuration and storage duration (year of manufacture) on the strain response of 3D-printed composite components. For the analysis, a methodology based on strain gauge measurements of cantilever beams produced via additive manufacturing from Onyx reinforced with continuous glass fibers was employed. Two sets of specimens, incorporating two reinforcement densities (2 and 4 layers) and distinct layup arrangements, were printed for experimental testing. The first set was stored for three years at 22 ± 2 °C and 35 ± 5% relative humidity, while the second set was printed immediately prior to testing. Quasi-static bending strains and fundamental natural frequencies were captured using surface-mounted strain gauges on the top and bottom surfaces of the beams. A comprehensive analysis of the results demonstrated that the proposed strain gauge-based experimental methodology did not confirm a dominant effect of stored conditions on the monitored mechanical characteristics. Conversely, the methodology successfully identified local strain asymmetry, which became evident upon a detailed comparison of experimental quasi-static and dynamic measurements. To evaluate computational strategies, four modeling approaches combined with analytical and numerical homogenization techniques were implemented. The results revealed that global homogenization continuum models overpredict bending strains due to averaging elastic properties across the cross-section. In contrast, explicit layered shell and 3D solid composite models provided good agreement with experimental measurements. The results confirm that the arrangement of reinforcement layers significantly influences the mechanical properties of the specimens.

## 1. Introduction

Fiber-reinforced composite materials (e.g., reinforced with carbon or Kevlar fibers) represent a key group of high-performance materials in modern engineering. By combining two or more constituents, a synergy of their properties is achieved, yielding high specific strength, stiffness, and excellent static and dynamic characteristics. Due to these properties, they find widespread application in aerospace, automotive engineering, civil engineering, and biomechanics [1].

With the development of modern manufacturing technologies, additive manufacturing of composites is gaining increasing prominence—for example, via Fused Filament Fabrication (FFF) using advanced matrices and reinforcement with short or continuous carbon and glass fibers. Additive manufacturing offers significant geometric freedom along with new capabilities for local fiber orientation and reinforcement stacking. The mechanical response and flexural properties of these printed composites depend heavily not only on the fiber volume fraction itself, but notably on the reinforcement layup sequence, infill type and density, as well as the even or odd number of layers. Although FFF technology can exhibit a higher occurrence of internal voids compared to conventional manufacturing processes, this reduction in properties is often offset by the ability to precisely orient fibers into the most highly stressed regions [2,3]. The specific characteristics of conventional 3D printing versus additive manufacturing enhanced by Continuous Fiber Reinforcement (CFR) technology are summarized in [4].

**Figure 1:**
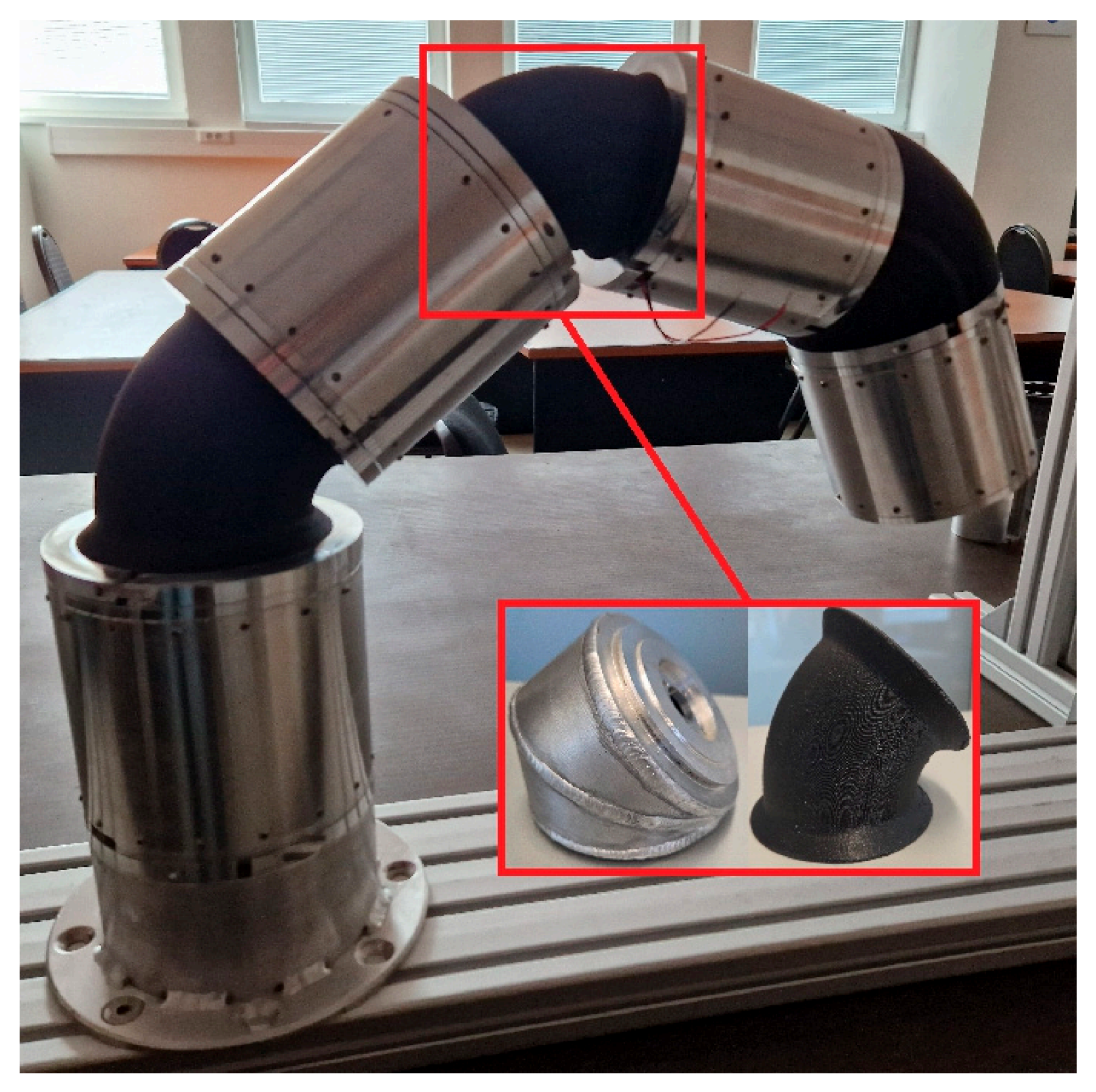
Demonstration of a physical model of a rotational joint made of aluminum alloy alongside its 3D-printed alternative, maintaining comparable mechanical properties.

CFR technology, utilized by the Markforged^®^ Mark Two system [5], enables the deposition of continuous fibers (carbon, glass, or Kevlar) directly into a matrix during the printing process, thereby achieving a significant increase in component strength [6,7]. The application of continuous fiber printing is promising, for instance, in the field of shape-optimization for complex components of robotic manipulators (Figure 1).

Non-destructive testing (NDT) methods, such as visual inspection, ultrasonic testing, thermography, radiography, electromagnetic testing, acoustic emission, and shearography, are used to evaluate internal integrity and detect defects without altering the physical properties of the parts [8]. While NDT methods primarily serve to uncover defects, precise strain measurement under defined loading is crucial for verifying mechanical properties and validating numerical models (e.g., via the finite element method—FEM). For this purpose, strain gauges are standardly employed in engineering practice. However, their application to additively manufactured composites presents specific challenges due to the inhomogeneous and anisotropic structure, as well as the risk of local stiffening at the measurement site in lower-modulus polymer matrices [9].

Previous studies on continuous fiber-reinforced 3D-printed composites have mostly examined the effect of fiber content, layup and infill parameters on the tensile, flexural or impact properties of freshly printed specimens [2,3], and have developed multiscale models of these materials. The use of strain gauges on printed polymers has been addressed mainly in terms of their local reinforcing effect [9]. The present study is a comprehensive experimental and numerical investigation of additively manufactured composite structures. The experimental part focuses on two independent questions. The first concerns the influence of the number and position of continuous glass-fiber layers on the quasi-static strain response and the first natural frequency of cantilever beams; this effect is evaluated within each specimen set and is therefore independent of the time elapsed between the sets. The second question concerns the stability of the response over time. For this purpose, two sets of nominally identical specimens, printed three years apart (2022 and 2025) on the same Markforged^®^ Mark Two printer [5] using identical printing parameters and material compositions, were compared; the first set was stored under laboratory conditions, whereas the second was tested immediately after printing. This comparison distinguishes the present work from the existing literature [3,10,11]. Since the sets differ not only in storage time but also in the date of the print run, the observed differences reflect the combined effect of aging and the process repeatability of additive manufacturing, which cannot be separated in the present study.

The experimental part is followed by the implementation of comprehensive numerical simulations using four modeling approaches combined with two distinct homogenization methods [12,13]. The results provide a methodical framework for selecting the optimal computational model for heterogeneous materials produced by additive manufacturing technologies, where classical homogenization theories fail due to the presence of processing defects, fiber orientation, and interlayer interface properties [14,15].

## 2. Materials and Methods

Composite specimens for this study were prepared using the CFR method on a Markforged^®^ Mark Two 3D printer (Markforged Inc., Waltham, MA, USA). The printer features a build volume of 320 × 132 × 154 mm, a *Z*-axis layer resolution of 100 µm and a precision-ground build plate. The composite materials utilized in this study were Onyx and HSHT (High-Strength, High-Temperature) fiberglass [16]. The Onyx filament consists of a nylon (PA6) matrix reinforced with chopped carbon fibers [17,18,19].

**Figure 2:**
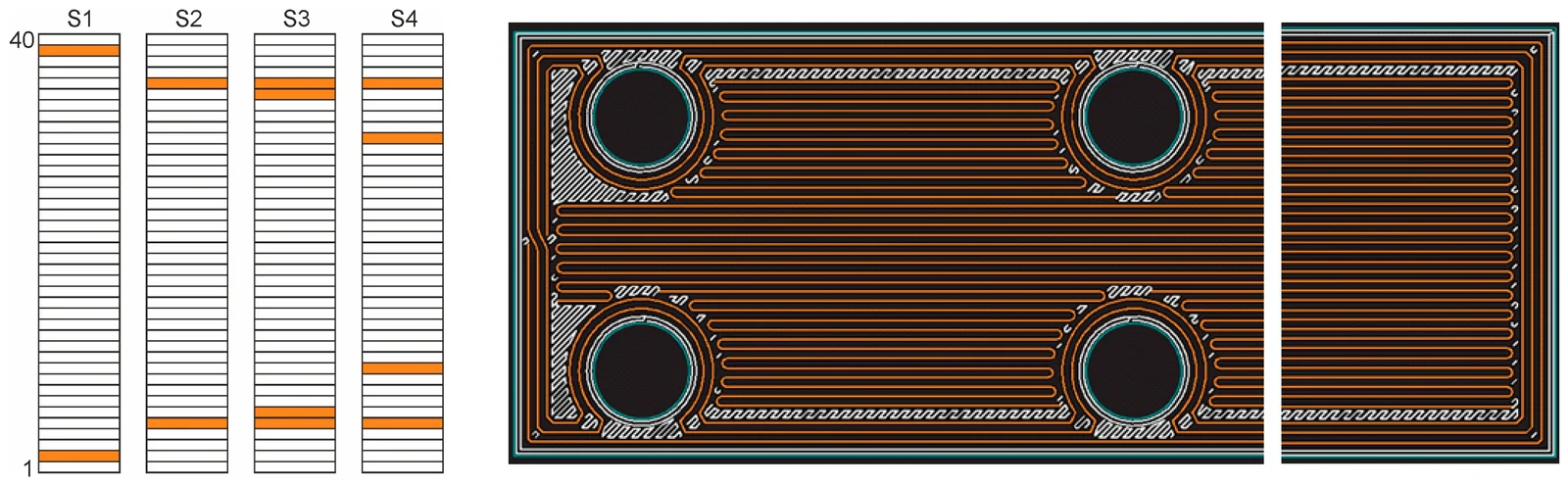
Printed specimens: (**a**) Position of reinforcing layers; (**b**) Arrangement of the HSHT continuous fiberglass in the layer 2 within specimen S1.

Based on the proposed nominal dimensions of 40 × 4 × 320 mm test specimens were fabricated in four different configurations (Figure 2a). Detailed information regarding the location and configuration of the HSHT fiberglass reinforcement layers within the Onyx matrix is summarized in Table 1. Layers are numbered sequentially from the build plate of the 3D printer upward. Each specimen consists of a total of 40 layers, which, at a nominal layer thickness of 0.1 mm, corresponds to a total cross-sectional height of 4 mm.

Specimens were prepared using a Markforged^®^ Mark Two 3D printer, utilizing Eiger^®^ slicing software (version April 2022, Markforged Inc., Waltham, MA, USA) to set the printing parameters and the toolpath of the continuous HSHT glass fibers within the Onyx matrix. The raster direction of Onyx alternated by 90° between the layers, i.e., ±45° to the longitudinal axis of the specimen. Figure 2b shows an example of the layer 2 printing for specimen S1. Three concentric fiber rings were deposited along the outer perimeter of each reinforced layer.

### 2.1. Experiment

**Figure 3:**
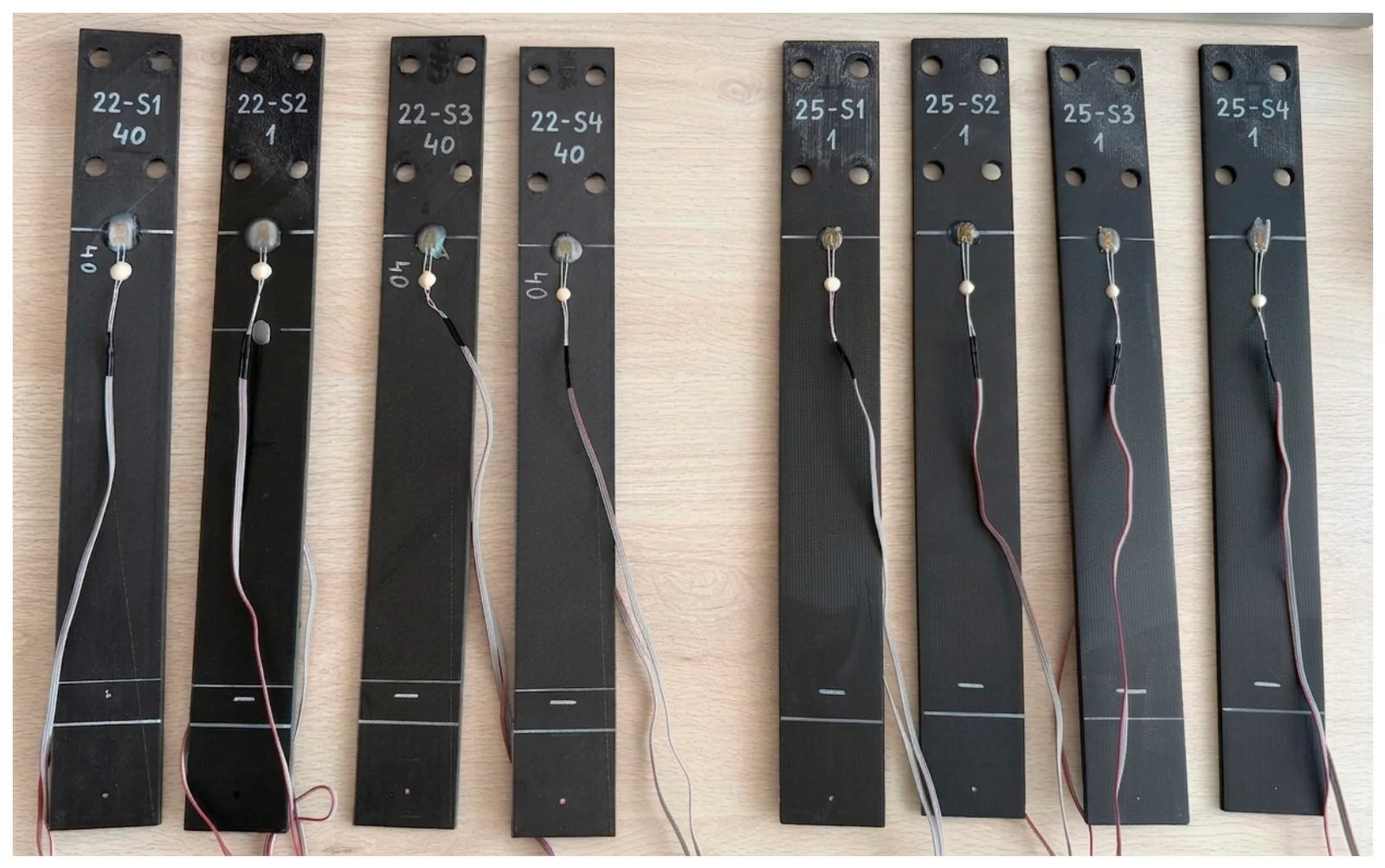
Tested specimen sets printed in 2022 and 2025 with bonded strain gauges.

The paper presents a comprehensive experimental analysis of the mechanical response of additively manufactured composite beams subjected to in-plane bending, with emphasis on the effect of reinforcement layer layout and count (Figure 2a) on their static and dynamic characteristics. Two specimen sets were prepared. The first set (22-S1 to 22-S4) was printed in 2022 and stored for three years under laboratory conditions at a room temperature 22 ± 2 °C and a relative humidity of 35 ± 5%, according to the laboratory records. After the mechanical tests, the moisture content of the 2022 specimens was determined by weighing before and after drying (24 h at 75 °C); the mass decreased by at most 1%. The second set (25-S1 to 25-S4) was printed in 2025 and tested immediately after printing; its moisture content was not measured. Both sets were produced on the same printer from the same type of material, with the same print file (identical toolpaths), the same version of the slicing software and the same printer firmware. One specimen per configuration was printed in each set, i.e., eight specimens in total (Figure 3), and each specimen was tested five times, each test consisting of five loading cycles. The results of one representative test run (ST2) are evaluated in detail. This run was chosen because it represents the stabilized material response following the initial seating of the fixture, remaining free from transient gripping effects or acquisition anomalies. The remaining test runs gave consistent results, with the half-bridge strains differing by less than 4% from run ST2.

The study thus contains two independent comparisons. The effect of the number and position of the reinforcing layers was evaluated by comparing configurations S1–S4 within each set, which does not depend on the time elapsed between the sets. The change in the response over time was evaluated by comparing corresponding configurations between the sets (e.g., 22-S1 and 25-S1).

The proposed experimental methodology focused on strain gauge measurements under quasi-static loading in the elastic deformation, followed by determination of the first natural frequency using the Fast Fourier Transform (FFT). To ensure consistent boundary conditions for both quasi-static and dynamic testing, experiments were conducted on a cantilever beam configuration.

**Figure 4:**
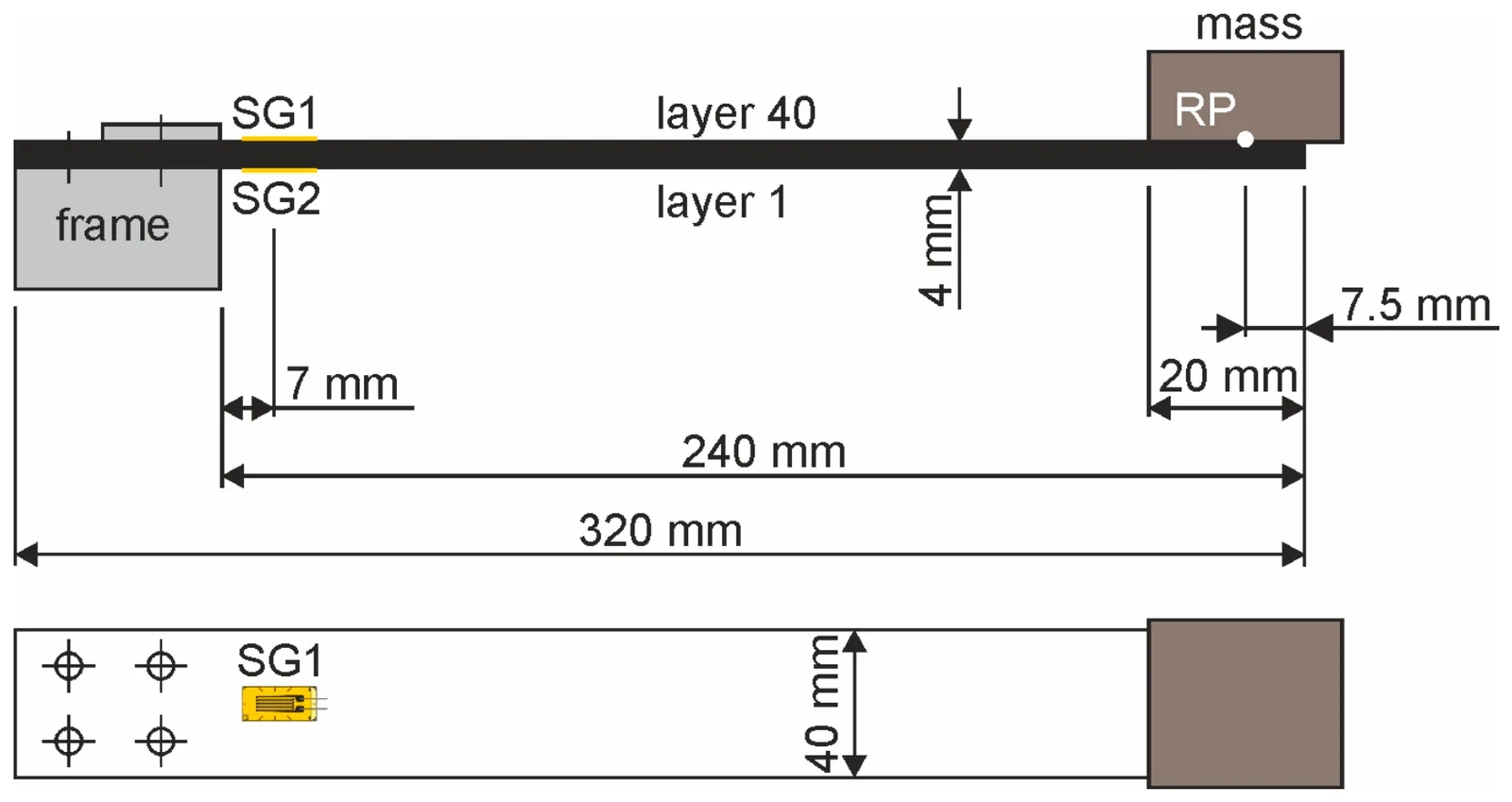
Schematic diagram of the cantilever beam setup, including nominal dimensions, strain gauge locations, and point of load application.

Figure 4 illustrates the schematic of the specimen boundary conditions, the location of strain gauges SG1 and SG2, and the load application point at the free end of the beam. A uniform specimen orientation was maintained for all measurements—the first printed layer (in contact with the print bed) was oriented downward, and the last printed layer upward.

**Figure 5:**
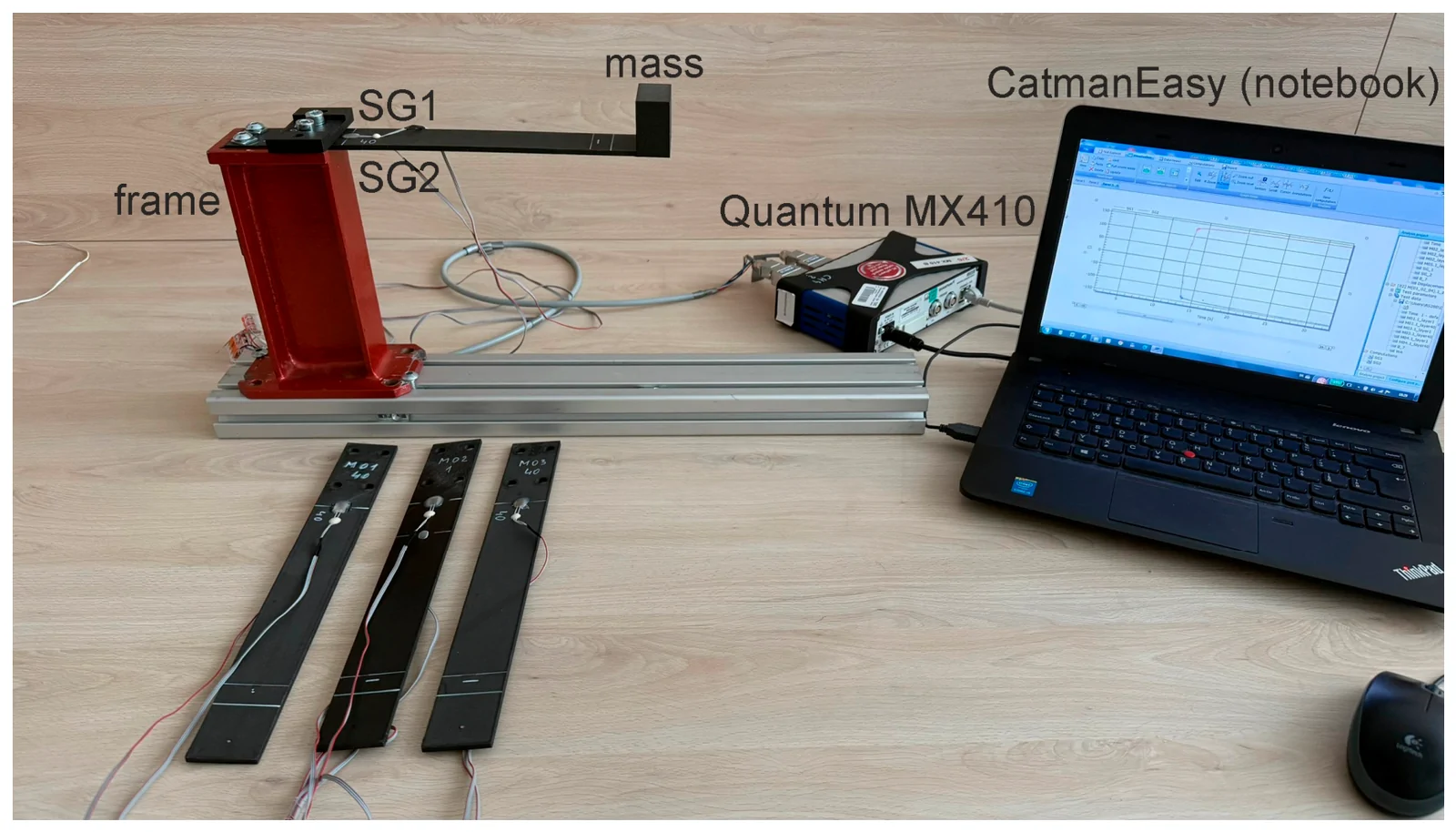
Overview of the experimental measurement chain and data acquisition setup.

The beam was clamped between a steel frame (from below) and a plastic plate (from above) fastened by four screws. During the measurement procedure, strain gauge SG1 was mounted on the top surface of the cantilevered beam, subjecting it to tensile loading under the applied load (m = 40.21 g) placed at the free end (Figure 5). Consequently, the strain data recorded by SG1 captured the longitudinal extension of the outermost fibers, yielding positive strain values. Conversely, SG2 on the bottom surface recorded compressive strain corresponding to fiber shortening.

The experimental measurements were structured into two operational stages:

- Quasi-Static measurements: The first stage aimed to characterize the strain responses of the composite specimens within their linear-elastic domain. During each test, loading cycles consisting of 10 s in the unloaded state and 10 s under load were repeated 5 times. This cyclic routine was employed to evaluate the strain elasticity, elastic recovery, and potential offset drift from the initial zero reference. In the final testing phase, the beam was subjected to a continuous load for 60 s, followed by a 60 s unloaded period. This step enabled the assessment of time-dependent strain responses in the composite material.
- Dynamic measurements: The second stage focused on determining the fundamental natural frequency by analyzing the free damped vibration response induced by an initial displacement impulse applied at the free end of the cantilever beam, repeated at 10 s, 20 s and 30 s of a recording of about 40 s. The first natural frequency was determined from the FFT of the SG1 and SG2 signals using an exponential window applied to each decay (derived from the three segmented excitation intervals), with a spectral interpolation step (zero-padded FFT) of 0.009 Hz. The ratio A_SG1_/A_SG2_ of the amplitudes of the spectral peaks of SG1 and SG2 was evaluated as well.

Experimental data acquisition and continuous signal logging were performed using a QuantumX MX410 DAQ system (HBM, Darmstadt, Germany). The strain gauges (type K-CLY4−0030−1−120−3−010-N, HBM) were connected in a standard quarter-bridge connection, sampling at a frequency of 150 Hz. Sensor calibration, real-time signal conditioning, and raw time-history recording were controlled via CatmanEasy software (v3.5.1.48, HBM). Subsequent post-processing, digital filtering, and time-domain signal evaluations were conducted in MATLAB R2022b (MathWorks, Natick, MA, USA).

### 2.2. Numerical Analysis

The flexural behavior of the cantilevered composite beam was numerically analyzed using four conceptually different modeling approaches. The objective of the study was to comparatively evaluate the accuracy of the individual methodologies against experimental measurements.

Numerical modeling was performed using finite element analysis in Abaqus/CAE 2022 software (Dassault Systèmes, Paris, France). Boundary conditions corresponding to a cantilever beam setup were applied to all models (Figure 4). This ideal rigid clamp represents the upper bound of the stiffness and of the natural frequencies and allows the modeling approaches to be compared without introducing parameters that are difficult to determine, such as contact friction or the screw preload. The load was applied via a reference point (RP) positioned at a distance of 7.5 mm from the free end. This reference point was coupled to the nodes of the loaded area, which extended up to 20 mm from the free end, accurately reflecting the physical contact region of the dead weight application. The strain at the gauge position was evaluated as the average over the gauge area centered 7 mm from the clamped edge, at the top and the bottom surface; in the shell model, it was evaluated at the outer surfaces of the top and bottom layers. The material orientation is identical in all models: axis 1 (longitudinal direction) is along the beam axis, and axes 2 and 3 are the in-plane transverse and the thickness directions, respectively. Perfect bonding between the layers was assumed in all models; delamination and the properties of the interlayer interface were not modeled. The material properties (Table 2) were taken from the manufacturer’s data [16,17] and were not measured on the specimens. The specimens were printed flat, with the layers stacked in the thickness direction and the continuous fibers predominantly along the beam axis (Figure 2b). Since the bending strain of the slender beam acts in the plane of the layers, the interlayer properties have a negligible influence in the elastic range, and perfect bonding between the layers was assumed. The chopped carbon fibers in Onyx are oriented mainly along the deposition direction, which makes the printed material anisotropic. As the raster direction alternates by 90° between the layers (±45° to the beam axis), the stiffness along the beam axis is an average over both directions, and Onyx was approximated as an isotropic material with effective properties. The reinforced layers were modeled as made entirely of HSHT with the effective modulus of the printed continuous fiberglass (21 GPa), not that of pure glass. The properties of the printed material may vary with the moisture content, voids and the actual fiber content of the layers; these variations were not measured.

Mesh convergence was assessed on the strain at SG1 and on the first natural frequency. The mesh was refined until the change in both quantities between successive meshes was below 1%. A more comprehensive breakdown of each modeling strategy is presented below.

The first model employs an analytical homogenization framework incorporating the Rule of Mixtures (ROM), Halpin–Tsai, and Clyne formulations. Among these, the ROM approach represents the classical analytical tool for estimating the effective macro-mechanical properties of composite systems. This method operates under the simplifying assumption of material homogeneity—despite the inherently heterogeneous nature of the composite constituents—thereby enabling the prediction of equivalent mechanical parameters from the volume fractions and mechanical properties of the individual components (Table 2). Onyx was treated as an isotropic matrix with effective properties, neglecting the local orientation of the chopped carbon fibers. The homogenized constants were evaluated for fiber volume fractions of 5% and 10%.

Integrating the ROM with more sophisticated semi-empirical formulations, such as the Halpin–Tsai and Clyne models, enables a significantly refined prediction of the effective mechanical properties of the composite system. While ROM provides baseline longitudinal estimates, the Halpin–Tsai formulation incorporates microstructural factors including fiber geometry, aspect ratio, and spatial distribution within the matrix, thereby offering superior precision in calculating transverse material properties. Consequently, the longitudinal Young’s modulus *E_L_* was evaluated according to the expression:

(1) $$E_{L}=V_{HSHT}\cdot E_{HSHT}+V_{O}\cdot E_{O},$$

where *V_HSHT_* is the volume fraction of HSHT fiberglass, *E_HSHT_* is the Young’s modulus of the fiberglass, *V_O_* is the volume fraction of Onyx matrix and *E_O_* is the Young’s modulus of Onyx matrix. The major Poisson’s ratio *ν_LT_* was determined using the equation:

(2) $$\nu_{LT}=V_{HSHT}\cdot \nu_{HSHT}+V_{O}\cdot \nu_{O},$$

where *ν_HSHT_* and *ν_O_* denote the Poisson’s ratios of the fiberglass and the Onyx matrix, respectively. The ROM method assumes that the fibers are perfectly straight, parallel, uniform in diameter, and exhibit homogeneous mechanical properties. Furthermore, perfect interfacial bonding is assumed, ensuring equal longitudinal strain in both constituents [20,21]. To evaluate the transverse Young’s modulus *E_T_*, the Halpin–Tsai model was applied [22,23], expressing *E_T_* as:

(3) $$E_{T}=\frac{1+\xi \cdot \eta \cdot V_{HSHT}}{1-\eta \cdot V_{HSHT}}\cdot E_{O},$$

(4) $$\eta =\frac{\frac{E_{HSHT}}{E_{O}}-1}{\frac{E_{HSHT}}{E_{O}}+\xi },$$

where *ξ* is the reinforcing factor (geometry factor) that accounts for the arrangement and distribution of the reinforcing fibers. For the evaluation of the transverse Young’s modulus *E_T_*, it is assumed that the coefficient *ξ* = 2 [24]. Furthermore, transverse isotropy is assumed, such that *E_T_* = *E_T′_*. The out-of-plane transverse Poisson’s ratio *ν_TT′_* was subsequently estimated employing the Clyne analytical formulation [25]. Although this model does not directly incorporate explicit geometrical fiber morphology, it utilizes *E_T_* as a primary input parameter, thereby implicitly accounting for the structural layout and cross-sectional arrangement of the fibers. Consequently, the transverse Poisson’s ratio *ν_TT′_* is defined as:

(5) $$\nu_{{TT}^{\prime }}=1-\nu_{TL}-\frac{E_{T}}{3K},$$

where

(6) $$\nu_{TL}=\nu_{LT}\cdot \frac{E_{T}}{E_{L}},$$

(7) $$K={\left(\frac{V_{HSHT}}{K_{HSHT}}+\frac{V_{O}}{K_{O}}\right)}^{-1},$$

where *K_HSHT_* and *K_O_* denote the bulk moduli of the fiberglass reinforcement and the Onyx matrix, respectively. The Clyne analytical model typically predicts a Poisson’s ratio *ν_TT′_* exceeding the isotropic limit of 0.5. This phenomenon arises because deformation along the continuous fiber direction is heavily constrained by the high stiffness of the reinforcement, causing an increased Poisson contraction in the unreinforced transverse direction *T′* [25]. The in-plane shear modulus *G_LT_* was subsequently evaluated using the Halpin–Tsai formulation in a manner analogous to *E_T_*, expressed as:

(8) $$G_{LT}=\frac{1+\xi \cdot \eta_{G}\cdot V_{HSHT}}{1-\eta_{G}\cdot V_{HSHT}}\cdot G_{O},$$

where

(9) $$\eta_{G}=\frac{\frac{G_{HSHT}}{G_{O}}-1}{\frac{G_{HSHT}}{G_{O}}+\xi }.$$

Due to symmetry considerations, the identity *G_LT_* = *G_TL_* is satisfied. Finally, assuming transverse isotropy, the out-of-plane transverse shear modulus *G_TT′_* was evaluated according to the expression:

(10) $$G_{{TT}^{\prime }}=\frac{E_{T}}{2\left(1+\nu_{{TT}^{\prime }}\right)}.$$

The first FEM (Model 1) was discretized using 20-node quadratic brick elements with reduced integration (C3D20R) and a transversely isotropic material model with the homogenized constants (denoted as ROM in Table 3) with the axis of transverse isotropy (the fiber direction) along the beam axis. A fully homogenized continuum formulation was adopted, thereby bypassing the explicit modeling of the orientation and layup of individual fiber-reinforced layers. Based on a mesh convergence analysis, an in-plane element size of 1 mm was selected.

**Figure 6:**
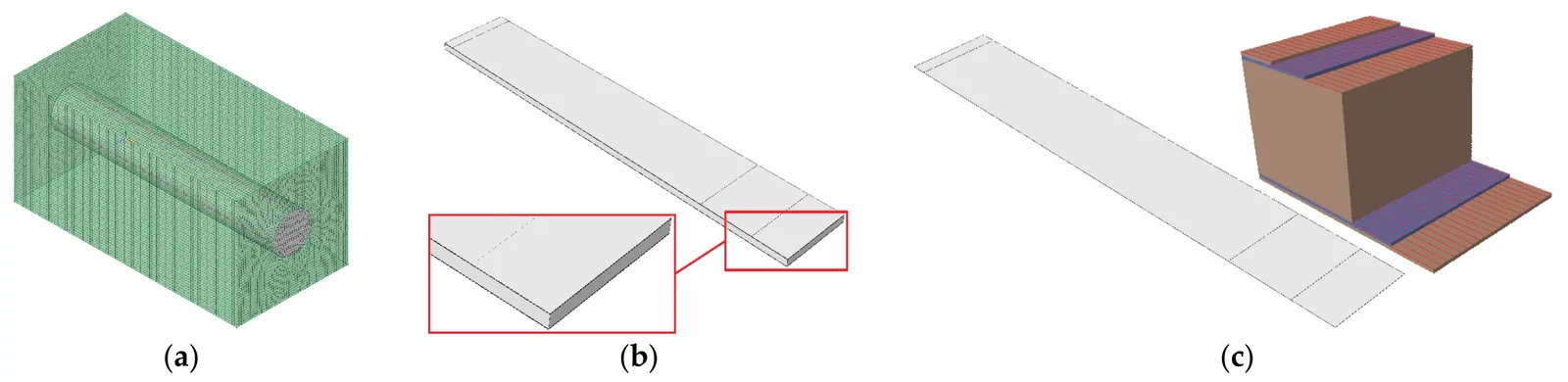
Overview of the finite element models: (**a**) Representative volume element (RVE) model; (**b**) Composite model; (**c**) Shell model.

The second modeling approach employs a numerical homogenization technique based on a Representative Volume Element (RVE). Previous findings [26] demonstrate that the RVE method yields results comparable to asymptotic homogenization (differing by merely 1% in predictive accuracy) while outperforming analytical homogenization schemes in predicting transverse elastic constants. This methodology relies on the assumption of a periodic fiber distribution, enabling the evaluation of effective material parameters via finite element analysis (FEA) over a reduced spatial domain [27]. The entire workflow—encompassing geometry generation based on the target volume fraction, spatial discretization, and FE simulations—was executed using Ansys Material Designer v2024 R1 (ANSYS, Inc., Canonsburg, PA, USA) (Figure 6a). The evaluated effective constants are summarized in Table 3. Analogously to Model 1, the second FEM (Model 2) retains the same geometry, mesh discretization, and continuum formulation, including the material orientation, but incorporates the homogenized constants derived via the RVE approach (Table 3).

The third FEM (Model 3) incorporates a fully layered 3D solid composite structure (Figure 6b), where individual layers are geometrically modeled and assigned their respective material properties (Table 2). The reinforced layers were modeled as isotropic with E = 21 GPa and the remaining layers as isotropic Onyx. Discretizing this layered geometry with C3D20R elements involved the highest mesh complexity owing to the extremely thin layer thickness. Nevertheless, the optimal mesh parameters were determined through a systematic convergence study, ensuring a favorable balance between element aspect ratio and computational efficiency. One element per layer was used through the thickness, with an in-plane element size of 1 mm.

The fourth FEM (Model 4) utilized a layered shell formulation (Figure 6c). To efficiently capture the flexural behavior, the structure was discretized using 8-node quadratic shell elements with reduced integration (S8R), with the ply layup defined according to the actual specimen structure. Each layer within the shell section was assigned the same material properties as in Model 3 (isotropic HSHT for the reinforced plies and isotropic Onyx for the remaining plies). This strategy follows conventional composite modeling practices, where each individual lamina is explicitly represented as a discrete layer. Based on the mesh convergence analysis, an in-plane element size of 1 mm was adopted, utilizing five integration points through the thickness of each layer.

The aim of the numerical part was to create the models as simply as possible, using only the data available to the user of the printer, i.e., the nominal volumes from the slicing software (Table 1) and the manufacturer’s material data (Table 2). The actual amount of fiber in a printed layer is difficult to estimate, because it is affected by voids, compaction of the filament and the toolpath. The reinforced layers were therefore modeled as made entirely of HSHT over the full layer thickness (Models 3 and 4), which corresponds to a fiber volume fraction of the whole specimen of 5% for S1 and S2 (2 of 40 layers) and 10% for S3 and S4 (4 of 40 layers). The same fractions were used in the homogenized models (Models 1 and 2), so that all four models contain the same amount of reinforcement and differ only in its distribution over the cross-section.

## 3. Results and Discussion

This paper presents the experimental results for specimens designated S1 through S4, which differ in the number and layup configuration of fiberglass layers while maintaining symmetry with respect to the cross-sectional neutral axis.

**Figure 7:**
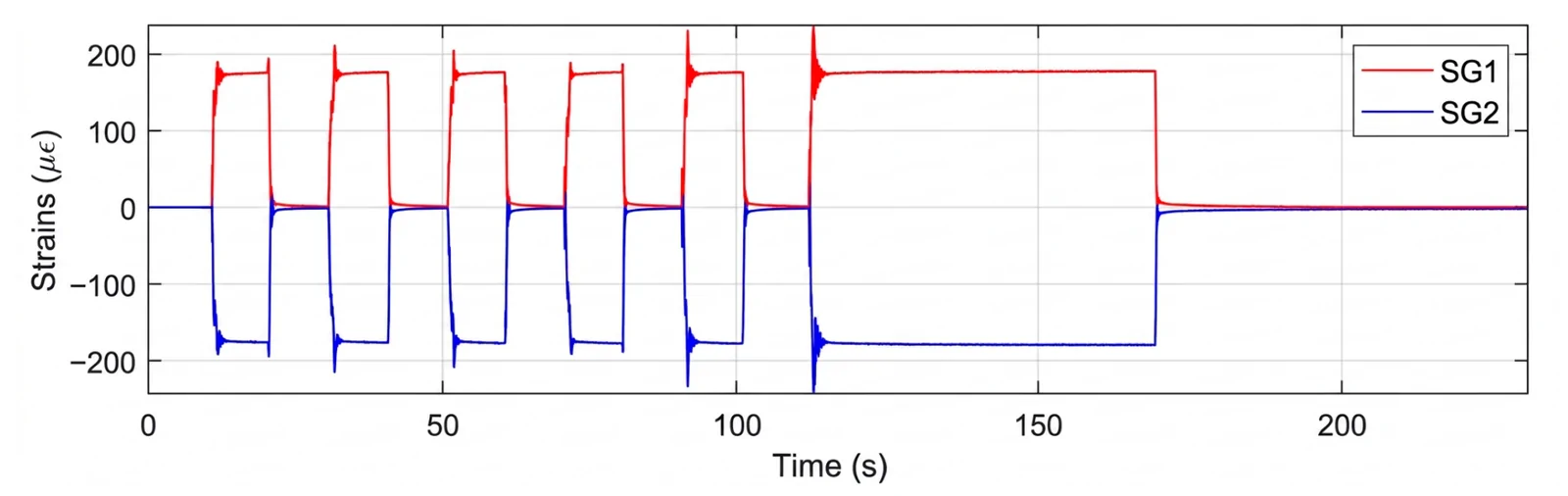
Time-dependent strain response of the 25-S1-ST2 composite specimen.

A representative time history of strains recorded under quasi-static loading conditions for specimen 25-S1-ST2 is shown in Figure 7. Notable transient spikes are observed at the onset of each load application cycle, which are attributed to dynamic inertial impact during manual placement of the weight onto the beam tip (human factor).

**Figure 8:**
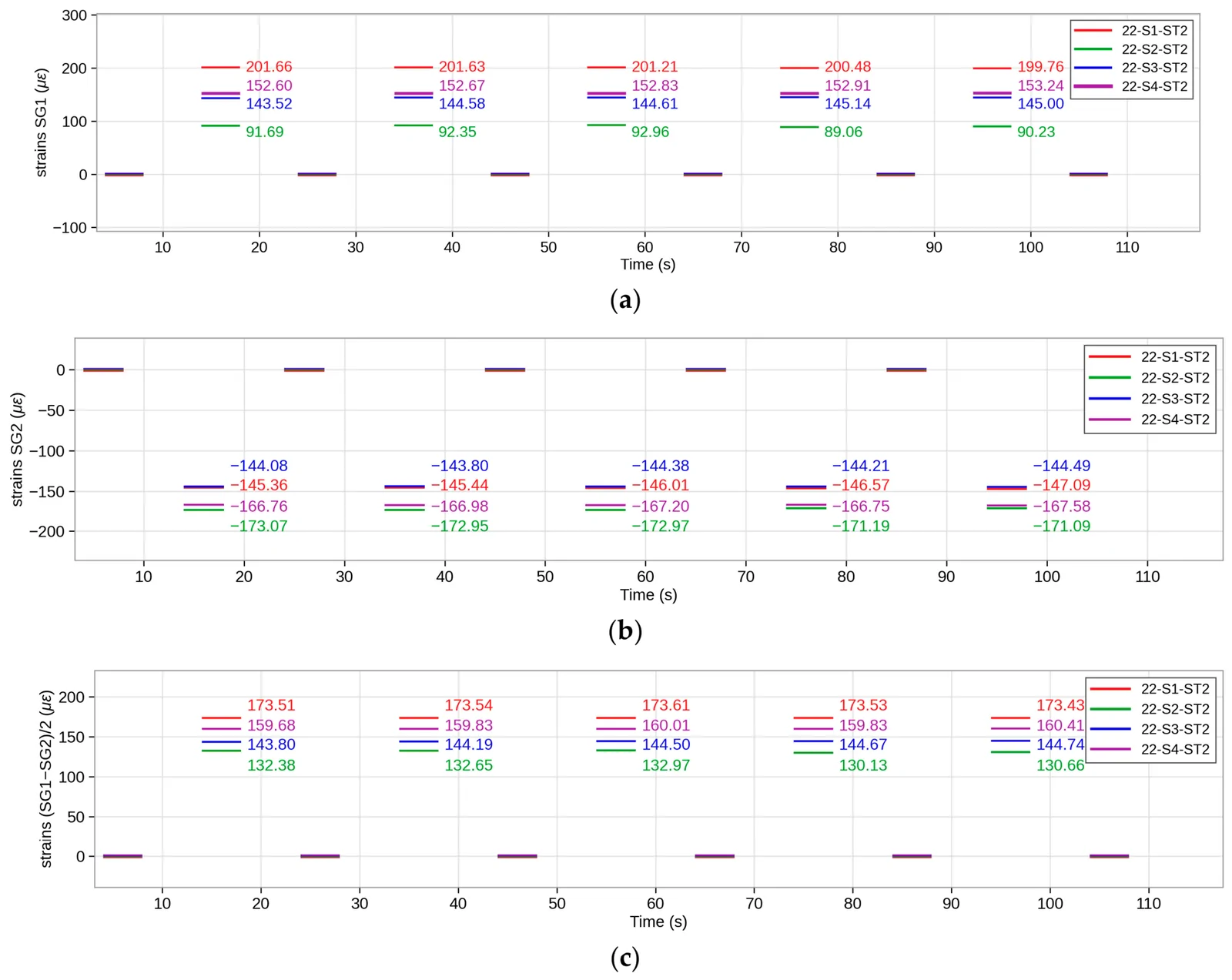
Time windows of 4 s showing mean values per interval—specimen set 2022: (**a**) Strains recorded by SG1; (**b**) Strains recorded by SG2; (**c**) Strains calculated as (SG1-SG2)/2.

**Figure 9:**
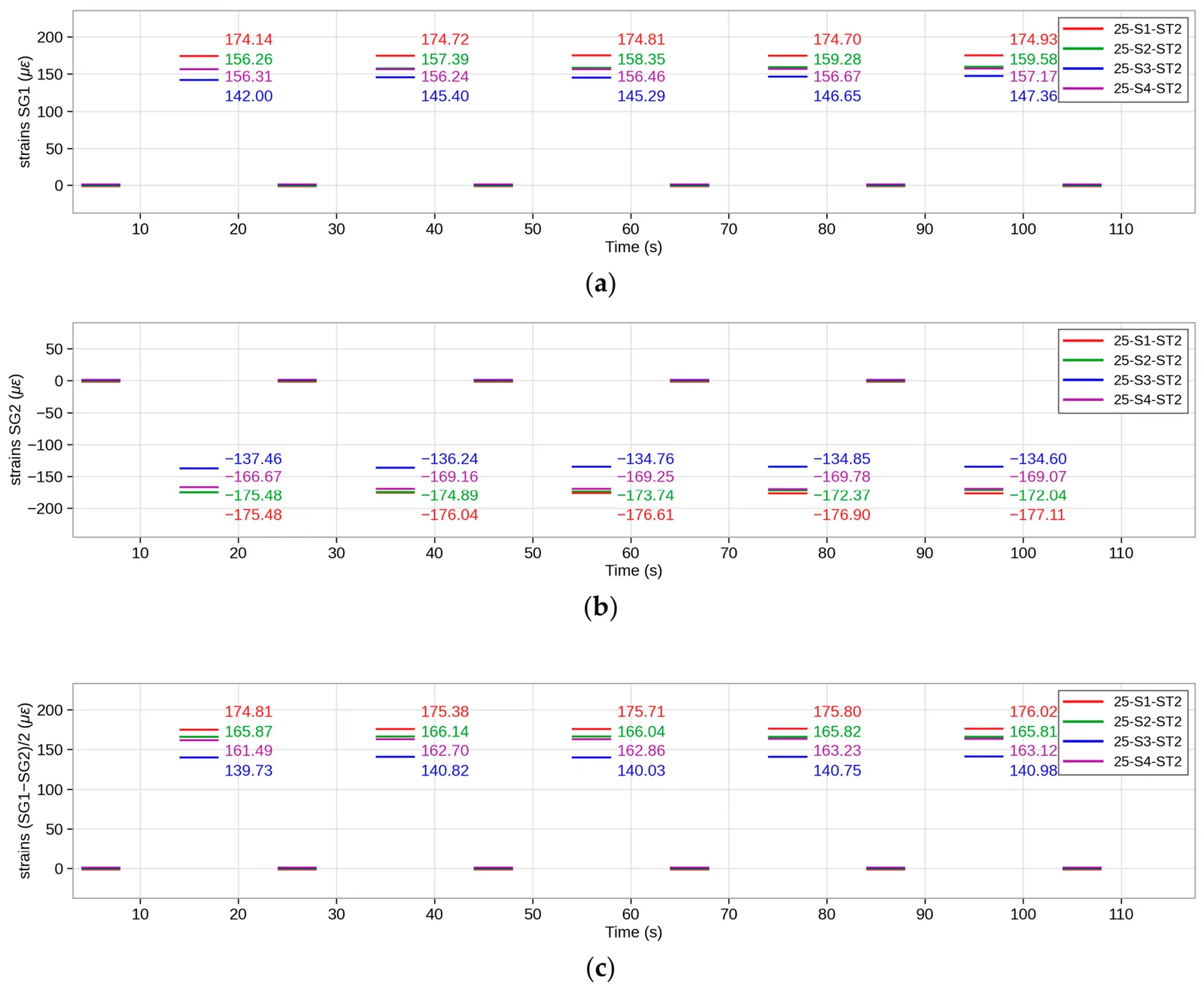
Time windows of 4 s showing mean values per interval—specimen set 2025: (**a**) Strains recorded by SG1; (**b**) Strains recorded by SG2; (**c**) Strains calculated as (SG1-SG2)/2.

To mitigate these dynamic transient effects during quantitative data extraction, four-second evaluation windows were selected (specifically 4–8 s, 14–18 s, 24–28 s, etc.), in which the transient oscillations had decayed, the strains are shown in Figure 8 (2022 set) and Figure 9 (2025 set). Figure 8a,b and Figure 9a,b present the strain responses measured by strain gauges SG1 and SG2 using a quarter-bridge connection. Furthermore, Figure 8c and Figure 9c document the strain values acquired via a half-bridge connection of sensors SG1 and SG2, which was implemented to isolate the pure flexural response by eliminating axial force components.

**Figure 10:**
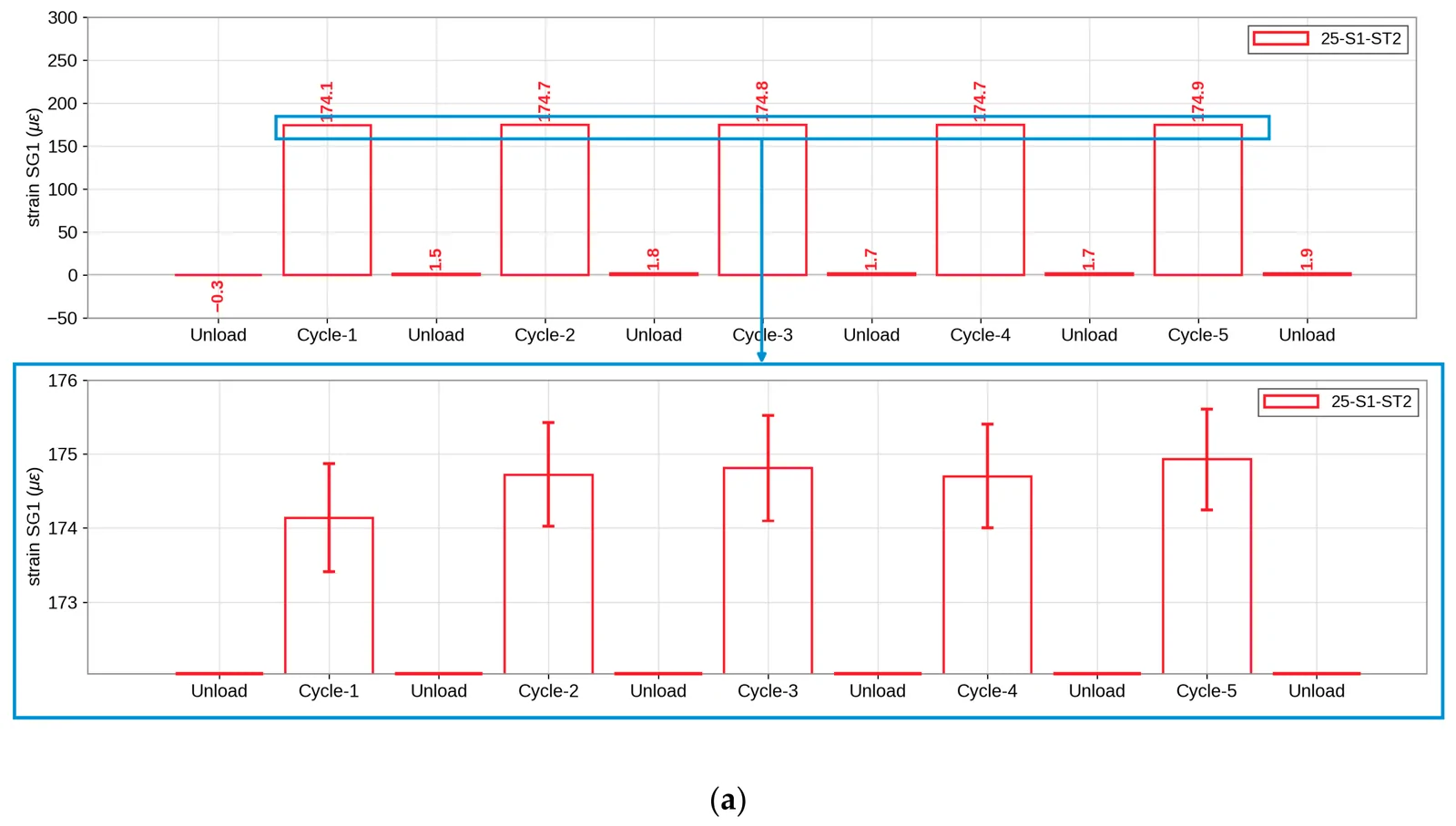

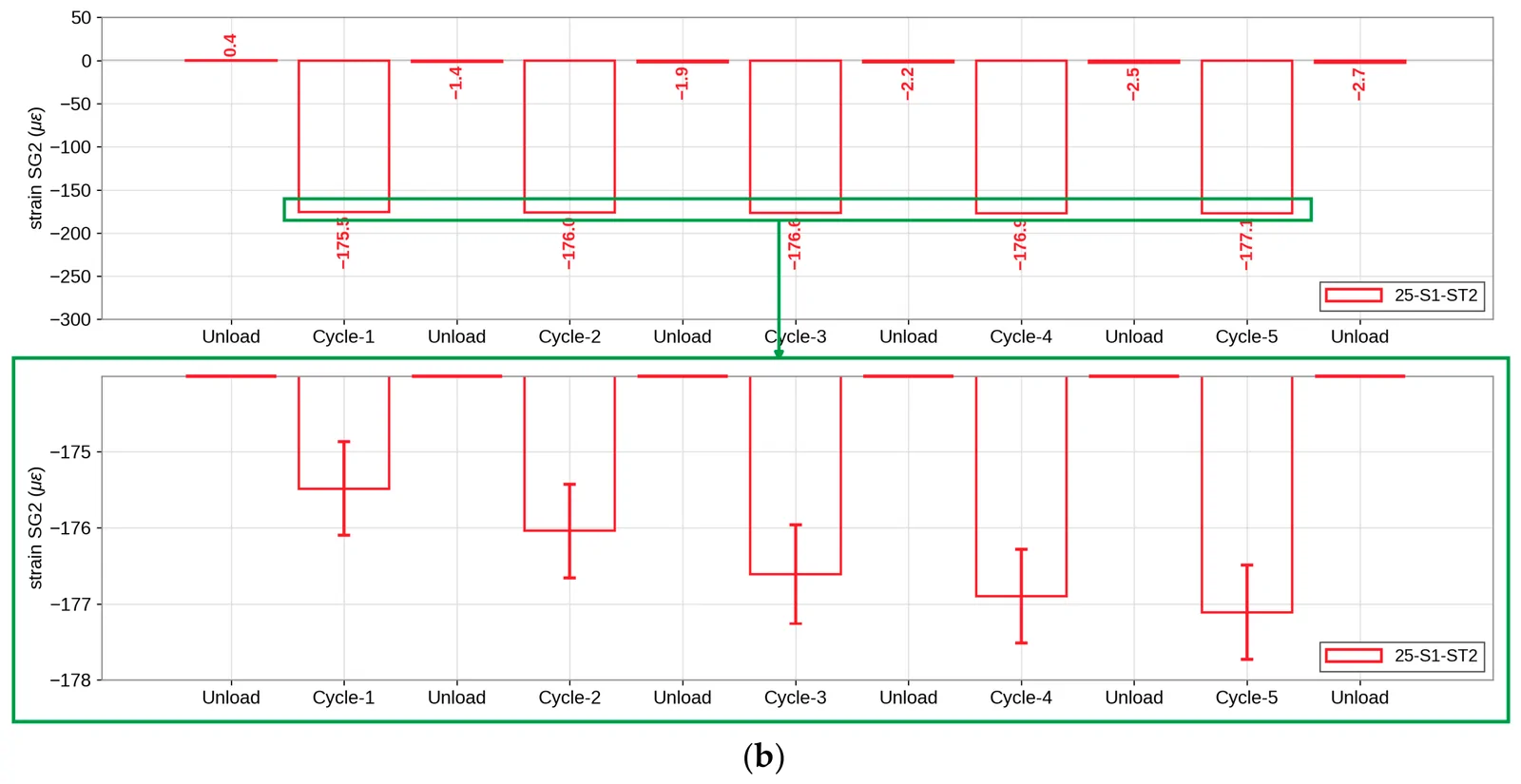
Mean strain values and their standard deviations calculated within the selected 4 s interval of each loading cycle for specimen 25-S1: (**a**) SG1 top surface sensor; (**b**) SG2 bottom surface sensor.

Figure 10 presents the mean strain values evaluated over the selected 4 s intervals. The error bars show the standard deviation calculated from the measured values within the selected 4 s interval of each of the five loading cycles of a single test run.

Analysis of the experimental data under quasi-static loading focused on comparing the magnitude of strains recorded by gauges SG1 and SG2 at the same cross-sectional location. The experimental strain measurements demonstrated high repeatability under quasi-static loading. During data analysis, an asymmetry in the absolute values of strains between the top and bottom fibers was identified. While several specimens exhibited the theoretical symmetry characteristic of pure bending, others displayed clear deviations, with strain magnitudes dominating either in the compressive (22-S2) or tensile (22-S1) region. The high repeatability of the measurements (Figure 8 and Figure 9) and the mirrored mounting of the strain gauges strictly rule out metrological errors, adhesive layer failure, or the effect of local stiffening by the sensor [9], as the symmetrical strain gauge arrangement would affect the overall cross-sectional stiffness uniformly.

As shown in Table 4 and Table 5, a significant divergence in strain predictions is observed between the simplified continuum models (ROM and RVE) and the explicit geometric layer models (Composite Layup and Shell Model). Both analytical (ROM) and numerical (RVE) homogenization methods overpredicted the bending strains for all specimens, by 16–80% (mean absolute error 36–37%). This substantial discrepancy stems from the fundamental limitation of global 3D continuum homogenization, which averages the elastic stiffness across the entire height of the cross-section. Consequently, these continuum models fail to capture the localized flexural contribution and the increased area moment of inertia provided by the fiber layers positioned near the outer surfaces. Since they depend only on the fiber volume fraction, they give identical strains for S1 and S2 (236–237 µε) and for S3 and S4 (188–189 µε). Conversely, the discrete composite layup model and the 2D layered shell formulation substantially lower errors. For S1, S3 and S4, their predictions deviated from the experimental half-bridge flexural strains by −6.0% to +2.5%, whereas for S2 they overpredicted the strain by 16.3% (2025 set) and 46.5% (2022 set). By explicitly defining the high-modulus HSHT fiberglass layers at their exact spatial coordinates, these explicit models resolve the bending stiffness of the heterogeneous cross-section, which is the expected outcome.

**Figure 11:**
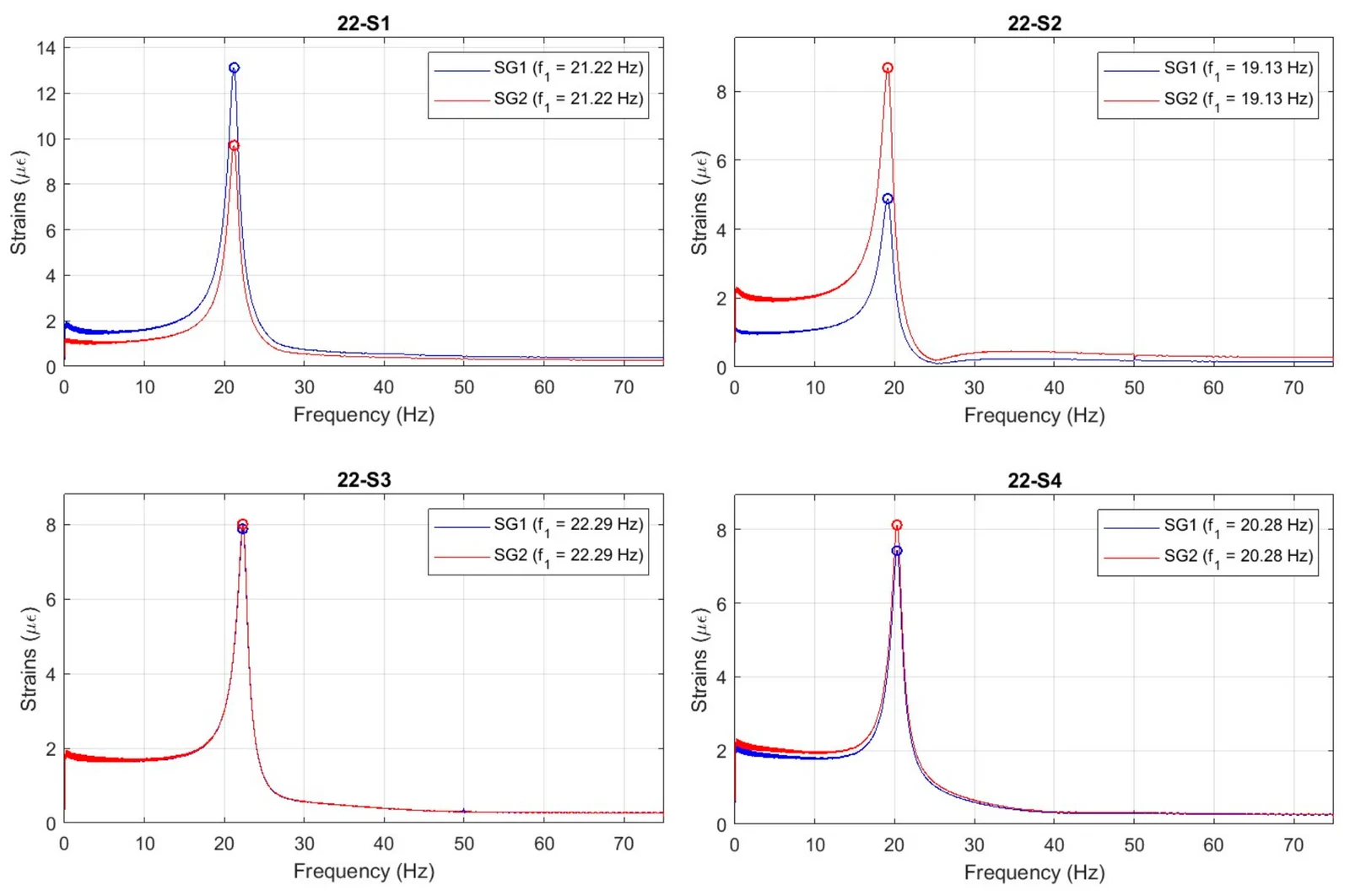
Frequency response spectrum obtained via Fast Fourier Transform (FFT) determining the fundamental natural frequency for the 2022 specimen set.

**Figure 12:**
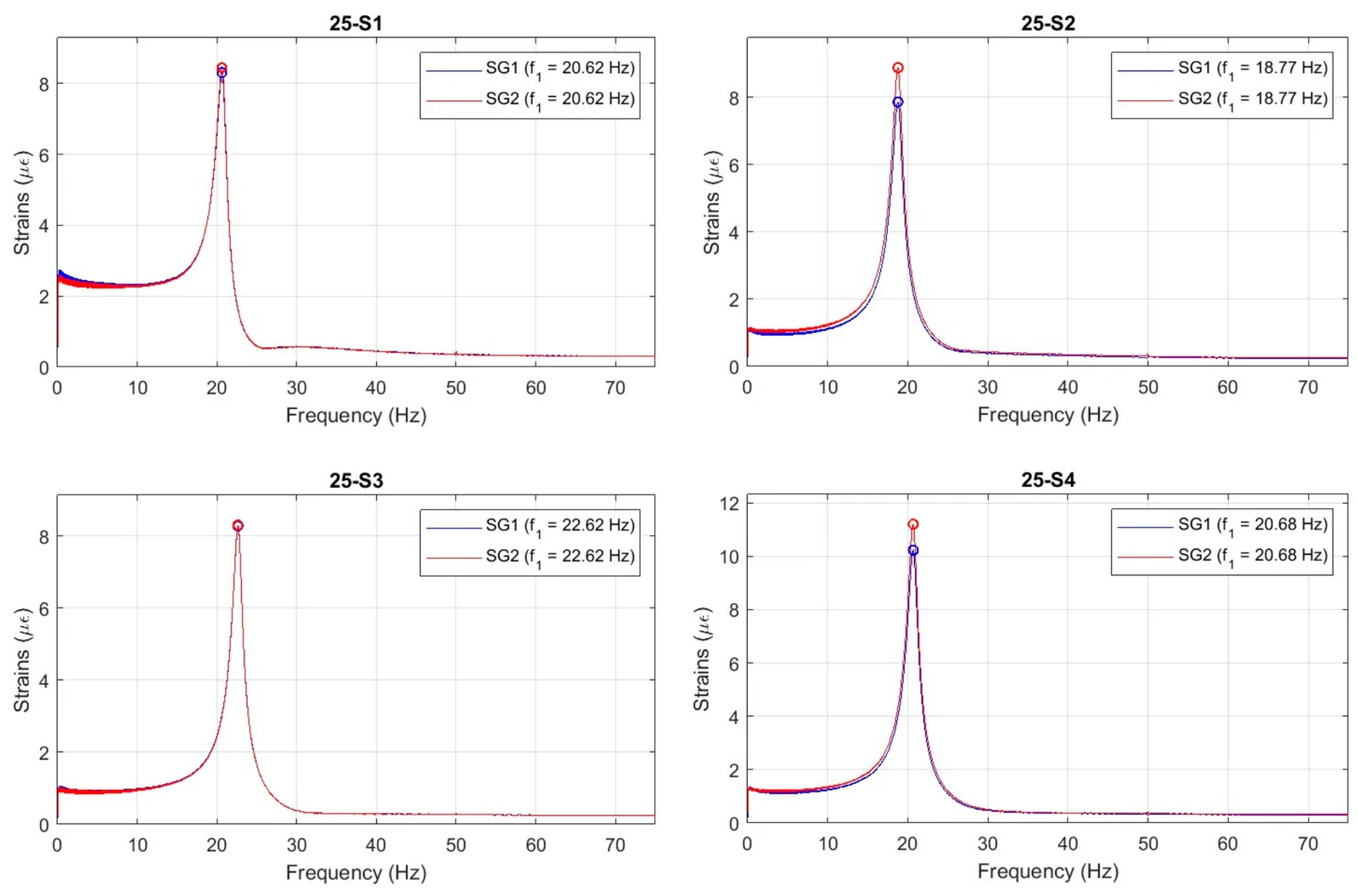
Frequency response spectrum obtained via Fast Fourier Transform (FFT) determining the fundamental natural frequency for the 2025 specimen set.

The subsequent modal analysis and dynamic signal processing via Fast Fourier Transform (FFT) provided key physical insights into the fundamental nature of the observed strain asymmetry. When extracting the fundamental natural frequency (*f*_1_) from the time-series recorded by the same strain gauge pairs employed during quasi-static testing, an exact match in fundamental natural frequencies was observed between the opposing sensors for each respective specimen (Figure 11 and Figure 12). Furthermore, clear variations in natural frequencies were identified across the different specimen configurations (Table 6). This behavior correlates directly with the distinct counts and stacking sequences of fiber reinforcement layers, which govern the global flexural rigidity of the composite cross-section.

Based on the results obtained from dynamic measurements (Table 6 and Table 7), the following key conclusions can be drawn:

1. Verification of dynamic integrity: The agreement between the natural frequencies captured by both sensors confirms that the dynamic response is governed by the global stiffness and mass distribution of the entire structure—properties that remain invariant across the component regardless of sensor location or surface orientation.
2. Confirmation of the local nature of strain asymmetry: The fact that sensors SG1 and SG2 recorded distinct strain magnitudes under static loading yet identified identical natural frequencies and a varying amplitude ratio (A_SG1_/A_SG2_) under dynamic excitation conclusively demonstrates that the observed static deformation asymmetry is exclusively local in nature. It stems from a localized neutral axis shift or local variability in elastic moduli within that specific cross-section which may be caused by internal porosity or by a small eccentricity of the reinforcing layers or fibers; however, it does not compromise the global integrity or dynamic rigidity of the additively manufactured beam. This phenomenon was clearly identified in specimens 22-S1 and 22-S2.
3. Identical amplitudes in symmetrical specimens: Specimens lacking internal cross-sectional asymmetry exhibited ideal, symmetric behavior under both static and dynamic conditions, as demonstrated by specimens 22-S3 and 25-S3.

These findings indicate that while quasi-static strain measurements on both surfaces are sensitive to local effects at the sensor position, whereas the first natural frequency reflects the global stiffness and mass of the beam.

These findings yield important methodological recommendations for strain-gauge testing of composite structures:

- Risk associated with reducing the number of sensors: To reduce experimental costs, strain gauges are sometimes applied to only one surface (e.g., solely on the side subjected to maximum tensile stress). However, if only a single sensor were used on the investigated specimens, internal inhomogeneity or local cross-sectional defects would remain completely undetected. Consequently, relying on a single sensor risks distorting the measured strain values and can lead to a misinterpretation of the material’s true mechanical behavior.
- Limitations of the standard half-bridge connection: In engineering practice, a half-bridge strain gauge connection is standard for evaluating planar bending because it automatically compensates for axial force variations (tension/compression) and parasitic torsional effects. However, due to the operational principles of the half-bridge connection, the recorded output reflects only the difference in strain. As a result, this setup conceals whether the absolute strain magnitudes on opposing surfaces are symmetrical or if asymmetry occurs due to a neutral axis displacement. The presented results show this critical methodological gap between actual sensor response and theoretical assumptions.

The homogenized models predicted identical frequencies for S1 and S2 and for S3 and S4. They underestimated the frequency of S1–S3 by up to 12.4% and slightly overestimated that of S4 (by up to 3.0%), whereas the layered models overestimated the frequency of all specimens by 4.3–9.9% and reproduced the ordering of the configurations. The mean absolute errors are comparable (5.5% for the homogenized and 7.3–7.5% for the layered models). The overestimation by the layered models may be related to the idealized rigid clamp (the compliance of the plastic plate and of the specimen under the clamp lowers the natural frequencies, whereas the strain at the gauge position, given by the bending moment of a statically determinate cantilever, is affected only marginally), to the mass of the gauges and cables, and to modeling the reinforced layers as made entirely of HSHT.

The rigid clamp in the models is an idealization, since the real beam was clamped between a steel frame and a plastic plate fastened by screws. The compliance of such a clamp, caused mainly by the deformation of the plastic plate and by the compression of the polymer specimen, together with micro-slip at the interfaces, lowers the natural frequencies, which agrees with the observed overestimation by the layered models. For a cantilever beam, the bending moment at the gauge position does not depend on the clamp compliance; however, since the gauge was placed only 7 mm from the clamped edge—near a region of local stress-concentration and transition zone of the support—may contribute to the observed discrepancies in the strain magnitudes.

## 4. Conclusions

This study focused on a comprehensive experimental investigation into the mechanical response of additively manufactured composite beams subjected to flexural loading, paying specific attention to the impact of reinforcement layer count and layup configuration on their static and dynamic behavior.

The presented experimental methodology combines quasi-static strain gauge evaluations within the linear-elastic range with modal analysis via Fast Fourier Transform. A novel element of this study is the comparison of two independent specimen sets manufactured on the same 3D printer with identical parameters, yet separated by a three-year production interval. The results therefore applies only to the tested storage conditions and do not prove the absence of aging. After three years of storage at 22 ± 2 °C and 35 ± 5% relative humidity, the strains of S1, S3 and S4 and the natural frequencies of all configurations differ by less than 3% from those of the freshly printed specimens, with no consistent sign; no systematic effect of storage was observed.

The quasi-static bending tests showed that some specimens exhibited the theoretically expected symmetry of the strains on the outermost surfaces, whereas several specimens exhibited marked asymmetry between the absolute strains on the top and bottom surfaces at the same cross-section. The high repeatability of the measurement makes a random error unlikely.

The dynamic measurements gave additional information on this asymmetry:

1. Agreement of fundamental natural frequencies: Frequency spectrum analysis revealed identical first natural frequency values derived from both strain gauges (SG1 and SG2) on each individual specimen (Figure 11 and Figure 12). This is expected, since the natural frequency is a global characteristic that depends on the total stiffness and the mass distribution of the beam and not on the position of the sensor; it therefore does not by itself indicate the absence of local defects.
2. Correlation of FFT amplitude spectra with static response: The ratio of the amplitudes of the spectral peaks of the two gauges mirrored the static strain behavior. Specimens with symmetric static strain fields displayed equivalent dynamic oscillation amplitudes across both sensors (e.g., specimen 25-S3, Figure 12). In contrast, specimens exhibiting static asymmetry yielded proportionally higher dynamic strain amplitudes on the gauge that recorded greater static strain levels (e.g., specimen 22-S2, Figure 11).

These results indicate that the asymmetry is local, i.e., related to the cross-section at the gauge position, and not to a change in the global stiffness of the beam. They are consistent with a local shift in the neutral axis. Possible causes are porosity, a deviation of the position of the layers or fibers, the bonding of the gauges and the clamping; clarifying them requires an examination of the internal structure, which is planned for further specimens in 2028.

The numerical comparison clearly indicates that classical homogenization theories (ROM, Halpin–Tsai, RVE) overpredicted the strains and cannot distinguish configurations with the same fiber content. The 3D solid and layered shell models gave practically identical results. For the studied configurations, the shell model is therefore preferable because of its lower computational cost.

## Figures and Tables

**Table 1:** Parameters of the printed specimens.

Properties	S1	S2	S3	S4
Materials	Onyx + HSHT	Onyx + HSHT	Onyx + HSHT	Onyx + HSHT
Layer height (mm)	0.1	0.1	0.1	0.1
Number of layers	40	40	40	40
Solid fill density (%)	100	100	100	100
Onyx nozzle temperature (°C)	252	252	252	252
Fiber nozzle temperature (°C)	275	275	275	275
Reinforcing layers	2, 39	5, 36	5, 6, 35, 36	5, 10, 31, 36
Concentric fiber rings	3	3	3	3
Onyx volume (cm^3^)	47.62	47.89	45.76	45.14
Fiber volume (cm^3^)	2.16	2.15	4.29	4.29
Nominal fiber volume fraction (%)	4.3	4.3	8.6	8.7

**Table 2:** Material constants used for the matrix and fiber components [16,17].

Material	Elastic Modulus (GPa)	Poisson’s Ratio (-)	Density (kg/m^3^)
Onyx	2.4	0.45	1200
HSHT fiber	21	0.22	1500

**Table 3:** Effective elastic constants of the composite material determined via analytical and numerical homogenization techniques.

Method	Fiber Volume Fraction [%]	E_L_ [GPa]	E_T_ [GPa]	E_T′_ [GPa]	v_LT_ [-]	v_LT′_ [-]	v_TT′_ [-]	G_LT_ [GPa]	G_TL_ [GPa]	G_TT′_ [GPa]	ρ [kg/m^3^]
ROM	5	3.33	2.67	2.67	0.44	0.44	0.54	0.925	0.925	0.867	1215
	10	4.26	2.96	2.96	0.43	0.43	0.58	1.03	1.03	0.934	1230
RVE	5	3.34	2.74	2.74	0.44	0.44	0.53	0.90	0.90	0.89	1215
	10	4.28	3.07	3.07	0.43	0.43	0.56	0.98	0.98	0.95	1230

**Table 4:** Summary of strains obtained from experimental measurements in half-bridge connection and numerical finite element simulations [με]. The experimental values are the mean ± standard deviation (SD) over the five loading cycles.

Specimen	Experiment 2022 (mean ± SD)	Experiment 2025 (mean ± SD)	ROM	RVE	Composite Layup	Shell Model
S1	173.5 ± 0.06	175.5 ± 0.47	237	236	165	165
S2	131.8 ± 1.28	165.9 ± 0.15	237	236	193	193
S3	144.4 ± 0.39	140.5 ± 0.55	189	188	144	144
S4	160.0 ± 0.28	162.7 ± 0.70	189	188	164	164

**Table 5:** Relative deviation of the numerical models from the experimental measurements in half-bridge connection = (model − experiment)/experiment.

Specimen	Experiment [με]	ROM [%]	RVE [%]	Composite Layup [%]	Shell Model [%]
22-S1	173.5	36.6	36.0	−4.9	−4.9
25-S1	175.5	35.0	34.4	−6.0	−6.0
22-S2	131.8	79.9	79.1	46.5	46.5
25-S2	165.9	42.8	42.2	16.3	16.3
22-S3	144.4	30.9	30.2	−0.3	−0.3
25-S3	140.5	34.6	33.8	2.5	2.5
22-S4	160.0	18.2	17.5	2.5	2.5
25-S4	162.7	16.2	15.6	0.8	0.8

**Table 6:** Summary of fundamental natural frequencies obtained from experimental measurements and numerical finite element simulations [Hz].

Specimen	Experiment 2022	Experiment 2025	ROM	RVE	Composite Layup	Shell Model
S1	21.22	20.62	18.58	18.61	22.13	22.29
S2	19.13	18.77	18.58	18.61	20.62	20.60
S3	22.29	22.62	20.83	20.88	23.81	23.79
S4	20.28	20.68	20.83	20.88	22.21	22.28

**Table 7:** Relative deviation of the numerical natural frequencies from the experiment and amplitude ratio A_SG1_/A_SG2_.

Specimen	Experiment [Hz]	Amplitude Ratio (A_SG1_/A_SG2_) [-]	ROM [%]	RVE [%]	Composite Layup [%]	Shell Model [%]
22-S1	21.22	1.35	−12.4	−12.3	4.3	5.0
25-S1	20.62	0.98	−9.9	−9.7	7.3	8.1
22-S2	19.13	0.56	−2.9	−2.7	7.8	7.7
25-S2	18.77	0.89	−1.0	−0.9	9.9	9.7
22-S3	22.29	0.99	−6.5	−6.3	6.8	6.7
25-S3	22.62	1.00	−7.9	−7.7	5.3	5.2
22-S4	20.28	0.91	2.7	3.0	9.5	9.9
25-S4	20.68	0.91	0.7	1.0	7.4	7.7

## Data Availability

The raw data supporting the conclusions of this article will be made available by the authors on request.

## Abbreviations

The following abbreviations are used in this manuscript:

FFF	Fused Filament Fabrication
CFR	Continuous Fiber Reinforcement
NDT	Non-destructive Testing
FEM	Finite Element Method
FFT	Fast Fourier Transform
ROM	Rule of Mixtures
RVE	Representative Volume Element
FEA	Finite Element Analysis
FE	Finite Element
